# Cytogenetic markers reveal a reinforcement of variation in the tension zone between chromosome races in the brachypterous grasshopper *Podisma sapporensis* Shir. on Hokkaido Island

**DOI:** 10.1038/s41598-019-53416-7

**Published:** 2019-11-14

**Authors:** Beata Grzywacz, Haruki Tatsuta, Alexander G. Bugrov, Elżbieta Warchałowska-Śliwa

**Affiliations:** 1 Department of Ecology and Environmental Sciences, Faculty of Agriculture, University of the Ryukyus, Nishihara, Okinawa 9030213 Japan; 2 Institute of Systematics and Evolution of Animals, Polish Academy of Sciences, Sławkowska 17, 31-016 Krakow, Poland; 3 The United Graduate School of Agricultural Sciences, Kagoshima University, Korimoto, Kagoshima 890-8580 Japan; 4 Institute of Systematics and Ecology of Animals, Russian Academy of Sciences, Siberian Branch, Frunze str. 11, 630091 Novosibirsk, Russia; 5 Novosibirsk State University, Department of Natural Sciences, Pirogov str. 2, 630090 Novosibirsk, Russia

**Keywords:** Cytogenetics, Entomology

## Abstract

The cytogenetic characteristics of the grasshopper *Podisma sapporensis* (two races 2n = 23♂ X0/XX and 2n = 22♂ neo-XY/neo-XX) were analysed through fluorescence *in situ* hybridization with rDNA and telomeric DNA probes, C-banding, fluorochrome and silver staining. For the first time, samples from the neighbourhood of a hybrid population (i.e., Mikuni Pass population) were studied. Our results indicated a significant degree of chromosomal differentiation between *P. sapporensis* races when comparing the number and position of the rDNA sites, as well as the heterochromatin composition and distribution obtained by C-banding and DAPI/CMA_3_ staining. Telomeric signals were usually detected at the distal and/or subdistal position of the autosomes; however, some chromosome ends lacked signals, probably due to a low number of telomeric repeats. On the other hand, telomeric DNA sequences were found as interstitial telomeric repeats in some autosomes, which can trigger a variety of genome instability. B chromosomes were found in specimens belonging to both main races from nine out of 22 localities. Four types of X chromosomes in the X0/XX race were identified. It was concluded that the physical mapping of rDNA sequences and heterochromatin are useful as additional markers for understanding the phylogeographic patterns of cytogenetic differentiation in *P. sapporensis* populations.

## Introduction

Chromosomal changes (e.g., peri- or paracentromeric inversions, mutual translocations) are considered to have played an important role in evolution, diversification and speciation^1–3^ because they could be a trigger for impeded gene flow between populations (e.g.)^4,5^. In contrast to expectations, there are still many studies that have raised some doubt with respect to the crucial role of chromosomal rearrangements on speciation^6^.

*Podisma sapporensis* Shiraki, 1910 (Orthoptera, Acrididae, Melanoplinae) is one of the most highly variable species with respect to karyotypes and it therefore provides an excellent opportunity to determine the role of chromosomal rearrangements in speciation^7–10^. This species is distributed in Hokkaido, southern Kuril Island (Kunashir Island) and Sakhalin. Comparative cytogenetic studies have shown that this species is represented by two major chromosomal races, X0/XX (2n = 23♂, 2n = 24♀) and neo-XY/neo-XX (2n = 22♂♀). Both races differ by Robertsonian fusion between the X chromosome and M5 autosome^7,11^.

During the last glacial period, *P. sapporensis* probably experienced population fragmentations into two main refuges on two sides of the central mountain system separated by the ridges of the Daisetsu and Hidaka Mountains, causing significant genetic differentiation for contemporary populations owing to the disruption of gene flow. The neo-XY/neo-XX chromosomal race might have arisen allopatrically during the process of such geological events and sequentially expanded in distribution in the eastern part of Hokkaido^7,12^. Further evolution of the X0/XX and neo-XY/neo-XX races led to the formation of several chromosome subraces in each of these races^7,8,10^.

So far, hybrid individuals between the X0/XX and neo-XY/neo-XX chromosome races have occasionally been found in the boundary between different chromosomal populations; however, the number of hybrid individuals in the natural environment is very few and no distinctive hybrid zone between the two races has ever been found. This suggests that some strong barriers are hampering hybridization, such as topographic factors and/or selection against the heterozygote^13^. Meanwhile, experimental hybridization between some chromosomal races has resulted in fertile hybrid progeny^8,14^, implying that the chromosomal rearrangements do not perfectly contribute to reproductive isolation. Despite this, zygotic reproductive barriers have also been found between the X0/XX race (i.e., Teine and Shimokawa populations)^8,10^ and the neo-XY/neo-XX chromosome race (i.e., Akan population). Therefore, in the case of *P. sapporensis*, these results suggest that the strength of the under-dominant effect depends on the combination of populations^10^.

The present study aimed to determine the chromosomal variability of *P. sapporensis* populations and identify differences in the locations of gene clusters amongst localities by means of several cytogenetic markers. The comparison was achieved by molecular (fluorescence *in situ* hybridization - FISH, with 18S rDNA and telomeric DNA) and classical (C-banding, silver impregnation and fluorochrome DAPI/CMA_3_ staining) cytogenetic methods. The principal objective was to ascertain: (1) how the individuals of *P. sapporensis* populations differed in the number and location of rDNA clusters; and (2) whether the combination of different cytogenetic markers led to a new facet of chromosomal differentiation compared to the standard cytogenetic markers alone.

## Results

### Physical mapping of 18S rDNA

**Figure 1:**
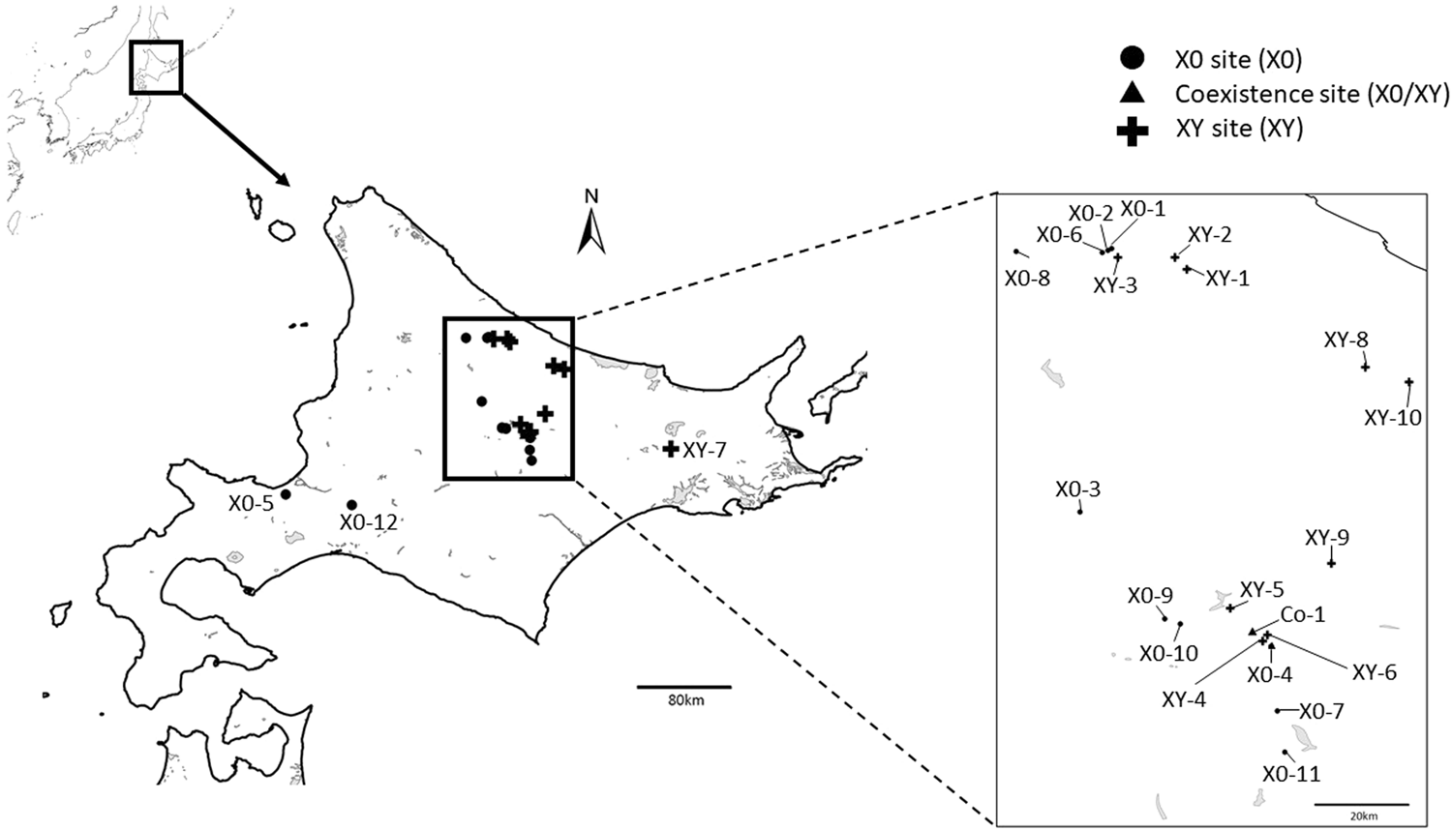
Geographic localities of *Podisma sapporensis* sampled in this study. Locality numbers are the same as in Table 1.

**Figure 2:**
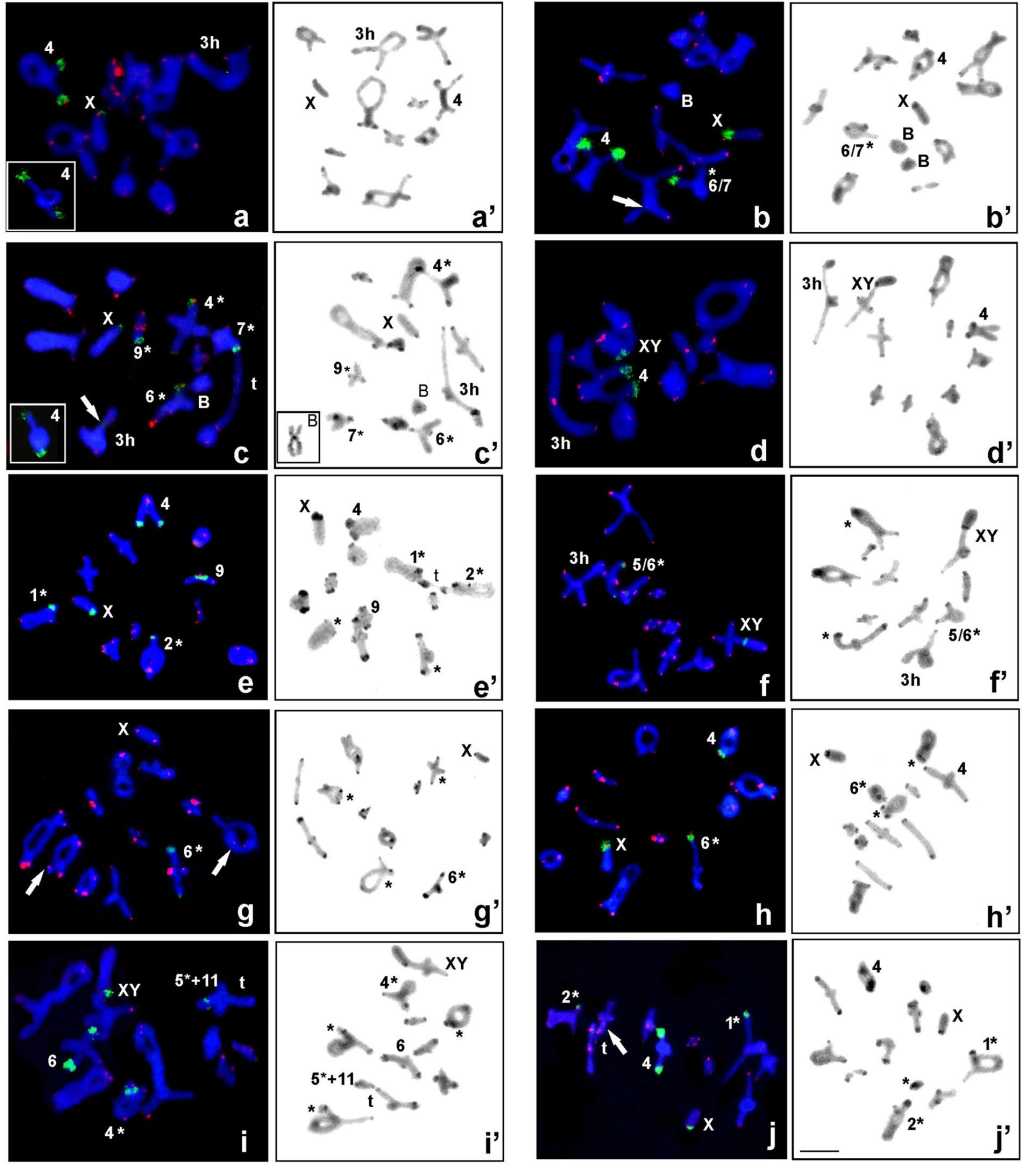
Examples of FISH with both 18S rDNA (green) and telomeric DNA (red) loci (**a**–**j**) and C-banding heterochromatin (**a**′-**j**′) of bivalents in diakinesis for the following *Podisma sapporensis*: X0-1,-2 (**a**–**c**,**a**′–**c**′) and XY-2 (**d**,**d**′); X0-3 (**e**,**e**′) and XY-4 (**f**,**f**′); X0-4 (**g**,**g**′), Co-1 (**h**,**h**′,**i**,**i**′); X0-10 (**j**,**j**′). The marked bivalent/s (number) and sex chromosome/s indicate the presence of rDNA clusters compared with the C-banding pattern; hybridization signals and the position of C-bands vary in size/position between homologous chromosomes (compare with Table 1) which are marked with an asterisk (*); (**a**,**c**) the insert in the left corner shows heterozygous M4 chromosomes forming a loop, indicating a partial absence of homology. B - indicates a metacentric supernumerary chromosome without rDNA probes (**b**,**c**) and C-bands (**b**′,**c**′); the insert in the bottom left corner (**c**′) shows this B chromosome in spermatogonial metaphase. 3 h - indicate heterozygous bivalent M3 (**a**,**a**′,**c**,**c**′,**d**,**d**′,**f**,**f**′); t - translocation between some chromosomes (**i**,**i**′,**j**). White arrows showed the presence of interstitial hybridization signals (ITSs) in long/medium autosomes (**b**,**c**,**g**) or during translocation between some of the bivalents (**j**). X/XY, sex chromosome/s. Scale bar = 10 µm.

Specimens from the vicinities of Nishi-Okoppe (Nishi-Okoppe-1 and -2) belong to the X0-Standard race (Table 1; Figs 1 and 2a,a′-c,c′). Other populations from the same region (Nishi-Okoppe-3,-4 and -5) belong to the XY/XX-Standard race (Table 1; Figs 1 and 2d,d′). In X0/XX karyotypes, the number of rDNA clusters ranged from two to five; they were located in the paracentromeric region of the medium bivalent (M4) and the X chromosome (Fig. 2a). The presence/absence of heteromorphism between homologous chromosomes in medium or small bivalents was a rare finding (Fig. 2b,c). Some samples were heterozygous or homozygous for the inversion on medium or small bivalents (based on C-bands) (Fig. 2a′–c′). On the other hand, in individuals belonging to the neo-XY/neo-XX race, only two high-intensity signals in the M4 chromosome pair and neo-XY bivalents were found (Fig. 2d).

**Table 1:** Geographic source, number of individuals of *P. sapporensis* analyzed in this study and summary of chromosomal data.

Locality number (no. indiv.)	Locality name	Latitude and longitude		Sex type X- morphology	Chromosome race	FISH		Pericentric inversion C-banding		M3 Hetero- zygotes	B type	Figs
		lat(hh) lat(mm)	long(hh) long(mm)			Heterozy- gotes	homozygotes + X (X0), neo-X (neo-XY), B	heterozygotes	structural homozy- gote			
X0-1(1)	Nishi-Okoppe-1	44 20.491	142 49.694	X0 X a thick	X0/XX-Standard		4, X			yes		4c
X0-1(2)				X0 X a thin	X0/XX-Standard		4, X			yes		2a,a′; 4c; 6a
X0-1(4)				X0 X sa-h	X0/XX-Standard		4, 6/7, X		e-6/7			
X0-1(5)				X0 X a thick	X0/XX-Standard	6/7	4, X	e-6/7			B_5iso_	2b,b′; 4c; 5b
X0-2(4)	Nishi-Okoppe-2	44 20.262	142 49.269	X0 X sa-h	X0/XX-Standard		4, X					
X0-2(9)				X0 X a thin	X0/XX-Standard	4, 6, 7,9	X	e-4, 6, 7, 9		yes	B_5iso_	2c,c′
XY-1(6)	Nishi-Okoppe-3	44 18.04	142 58.221	XY	XY/XX-Standard		4, neo-X					
XY-2(3)	Nishi-Okoppe-4	44 19.43	142 56.86	XY	XY/XX-Standard		4, neo-X			yes		2d,d′; 4f; 5e
XY-3(11)	Nishi-Okoppe-5	44 19	142 50	XY	XY/XX-Standard		4, neo-X			yes		
X0-3(5)	Kamikawa-1	43.833	142.767	X0 X a thick	X0/XX-Naganuma/Yotei	1, 2	4, 9, X	e-1, 2, 5	e-4, h-7			2e,e′; 4a,a′,d
X0-3(5)						4	X	e-4				
XY-4(3)	Kamikawa-2	43.583	143.117	XY	XY/XX-Tanno/Oketo	5, 6	neo-X	h-1, 2, 5, 6	e-4	yes		
XY-4(3)						5/6	neo-X	h-1, 5/6, e-4		yes		2f,f′
XY-5(2)	Kamikawa-3	43 38.815	143 3.238	XY	XY/XX-Tanno/Oketo	5, 6	neo-X	e-2, 4, 5, 6	e-1			
XY-5(3)						5, 6	4, neo-X	e-1, 2, 5, h-6	e-4	yes		
X0-4(1)	Mikuni Pass-1	43 34.762	143 7.772	X0 X a thin	X0/XX-Naganuma	6		e-2, 7, h-3	e-3			
X0-4(11)						6		e-2, 3, 5, 6				
X0-4(12)						6		e-2, 6, 8, e+h-4	e-3			2g,g′; 5a; 6b
X0-4(16)						6		e-1, 2, 6	e-3			
XY-6(3)	Mikuni Pass-2	43 35.687	143 7.587	XY	XY/XX-Tanno/Oketo	6, 7	neo-X	e-4, 6, h-7	e-1, 2, 5	yes		
XY-6(12)						4, 7	neo-X	e-1, 2, 4, h-7	e-3, 5		B_5iso_	
XY-6(15)						6, 7	neo-X	e-1, 2, 6, h-7	e-3, 5			4b,b′
Co-1(1)	Mikuni Pass-3	43 36.159	143 5.729	X0 X sa-e	X0/XX-Naganuma	6	4, X	e-6	e-1, 3, 4			2h,h′; 4e
Co-1(7)				XY	XY/XX-Tanno/Oketo		4, 5, neo-X	e-1	e-2, 4, 5	yes		
Co-1(11)						4	6, 5, neo-X	e-1, 2, 3, 4	e-5,6			2i,i′; 4g; 6c,d
Co-1(15)						4, 6	neo-X	e-1, 3, 4, 6	e-2,		B_5iso_	
Co-1(18)						4, 5	neo-X	e-1, 3, 5, 6	e-2			
X0-5(5)	Mt Teine	43.1	141.217	X0 X a thick	X0/XX-Standard		4, X, B				B_n_	3a,a′
X0-6(1)	Tempoku Pass	44 20.028	142 48.597	X0 X sa-h	X0/XX-Standard	4, 6/7	X	e-6			B_5iso_	
X0-6(6)						6	X	e-6				
X0-7(31)	Horoka Station	43 26.907	143 8.901	X0 X a thin	X0/XX-Standard	4, 6, 7	B	h-4, 6, 7			B_n_	3b,b′; 5b
X0-8(5)	Shimokawa	44 20.145	142 38.618	X0 X thick	X0/XX-Standard		4, X					
X0-8(7)						10,11	4, X, B	h-10, 11			B_n_	3c,c′
X0-9(6)	Kogen-spa	43 37.57	142 55.835	X0 X sa-e	X0/XX-Naganuma	1, 4, 5/6	2, X	h-1, 4, 9/10	h-2, e-5/6			5c
X0-10(2)	near Kogen-spa	43 36.986	142 57.643	X0 X sa-e	X0/XX-Naganuma	1, 2	4, X	h-1, 2, e-3				2j,j′
X0-11(1)	on the road to Upepe-sanke	43 22.125	143 9.77	X0 X sa-h	X0/XX-Naganuma		4, 6, X		e-6			5d
X0-12(2)	Maoi-spa	43 0.933	141 44.525	X0 X sa-h	X0/XX-Naganuma		4, 6/7, X	e-1	e-6			
X0-12(6)							4, X, B		e-6		B_n_	3d,d′
XY-7(1)	Teshikaga-A	43 27.636	144 14.605	XY	XY/XX-Standard		5/6, 7, neo-X			yes		
XY-8(4)	Maruseppu	44 43.733	143 143.25	XY	XY/XX-Tanno/Oketo	4, 5, 6	neo-X	e-2, 3, 4, 5, h-6				5f
XY-9(4)						4, 5	neo-X	e-1, 2, 4, 5		yes		
XY-10(17)	Hakuryu	44.083	143.4	XY	XY/XX-Tanno/Oketo		4, neo-X	e-1	e-3			

X type: a = acrocentric, thin = C-band restricted to the centromere, thick = C-band occupied the region next to the centromere, sa-e = subacrocentric with euchromatic short arm, sa-h = subacrocentric with heterochromatic short arm, a slash between two numbers indicates imprecise identification of the bivalent, Co-1 = coexistence site (X0/XY).

Samples from the Kamikawa-1 and Mikuni Pass-1 localities belong to the X0/XX-Naganuma/Yotei chromosome race whereas Kamikawa-2,-3 and Mikuni Pass-2,-3 belong to the XY/XX-Tanno/Oketo race (Table 1; Fig. 1 and 2e,e′-i,i′). In these individuals, regardless of the type of sex determination, one to five 18S rDNA loci were detected near the paracentromeric region in short euchromatic or heterochromatic arms in medium- and rarely long-sized autosomes, as well as generally in the sex chromosome/s (see below). Most of the studied samples exhibited heteromorphism in the presence/absence of rDNA clusters between homologous chromosomes (Table 1; Fig. 2e–i). Cytogenetic analysis of samples from the neighbourhood of Mikuni Pass comprising three populations (new data) and Kamikawa revealed that representatives of X0/XX and neo-XY/neo-XX coexist in this area. The C-banding revealed a high frequency of heterozygotes and homozygotes for the inversions in long and medium-sized autosomes (Fig. 2e′–i′).

**Figure 3:**
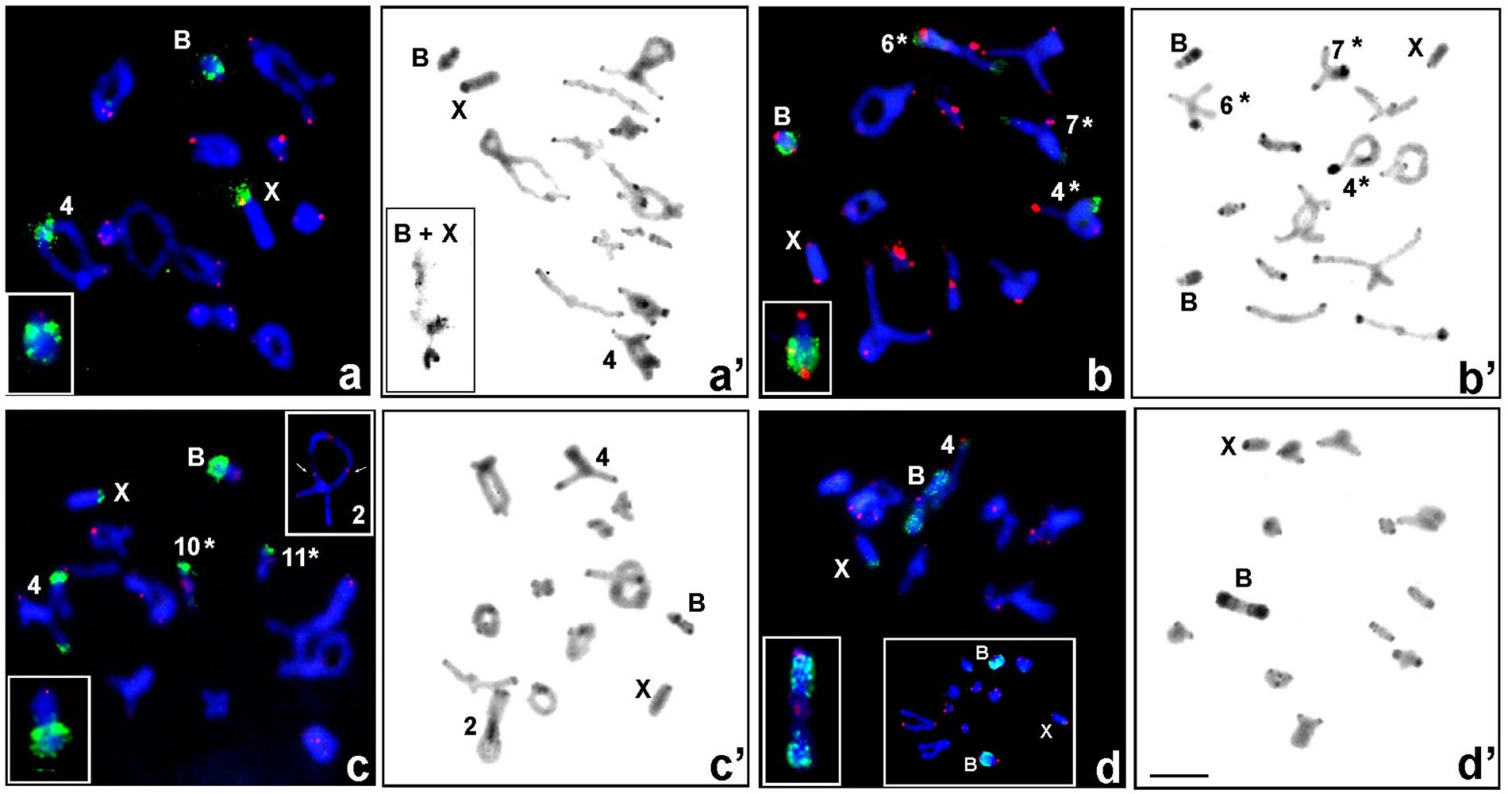
Meiosis cells with B chromosomes subjected to FISH with 18S rDNA (green) and telomeric DNA (red) probes (**a**–**d**), and C-banding (**a**′–**d**′) show some differences between individuals: X0-5 (**a**,**a**′), X0-7 (**b**,**b**′), X0-8 (**c**,**c**′) and X0-12 (**d**,**d**′). In diakinesis, Bs shows differences in the intensity of the hybridization signal (**a**–**d**; an enlarged image in the insert in the bottom left corner); early diplotene, Bs connected with the X [in the bottom left corner (**a**′)]; two (**b**′) or one univalent/s with different patterns of heterochromatin (**c**′); Bs bivalent in diakinesis with rDNA cluster [in the bottom left corner (**d**)] and two univalents in metaphase I [bottom panel in the middle (**d**)] and the heterochromatin pattern (**d**′). Additionally, some bivalent/s and sex chromosomes indicate the presence of rDNA clusters and C-bands; the hybridization signals and position of C-bands vary in size/position between homologous chromosomes (compare with Table 1) which are marked with an asterisk (*); in the insert on the top of the right corner, the arrows indicate ITSs on the L2 bivalent (**c**). Scale bar = 10 µm.

Inter- and intraspecific differences in the number of 18S rDNA hybridization signals in different-sized autosomes and sex chromosome/s were also observed in samples from other localities around Nishi-Okoppe, Kamikawa and Mikuni Pass (Hakuryu, Horoka Station, Kogen-spa, Maoi-spa, Maruseppu, Mount Teine, Shimokawa, Tempoku Pass, Teshikaga-A, Upepe-sanke) (Table 1; Figs 1 and 2j,j′). Analysis of these individuals showed variation in the position of 18S rDNA located on two to four bivalents, sex chromosome/s and in one type of B chromosome (see below) as well as polymorphism in two chromosome arms clearly seen in C-banding cells (Table 1; Figs 2j,j′ and 3a,a′-d,d′).

### Physical mapping for telomeric DNA repeats

Following FISH with the (TTAGG)_*n*_ probe (tDNA-FISH) using spermatogonial nuclei in meiosis (diplotene, diakinesis), signals were detected at the distal and/or subdistal position of the autosomes. Accordingly, not all chromosome ends showed hybridization signals in long bivalents (e.g., Fig. 2b,g). On other hand, near typical telomeric signals, interstitial hybridization signals (ITSs) in some of the long/medium sized autosomes (Figs 2b,c,g and 3c) or translocation between some of the bivalents (Fig. 2j) were observed.

### Supernumerary chromosomes and translocation between Bs and autosomes

B chromosomes (representing supernumerary elements with respect to the A standard chromosome set) were found in nine specimens belonging to both main races from nine out of the 22 localities analysed in this study using the FISH method (Table 1). In the present paper, it is likely that only one of the seven types of B chromosomes that have been described to date in *P. sapporensis* (see Fig. 1 in^15^) was found. No rDNA cluster was revealed in the metacentric B_5,6iso_ variant^15^, characterized by heterochromatin with very thin C-bands in both arms (Fig. 2b,b′,c,c′). In contrast, in four males from four populations belonging to the X0/XX race, acrocentric B_n_ types (probably new or heteromorphic B_1_ and B_2_ types) with a large cluster of 18S rDNA were detected (Table 1; Fig. 3a–d). They were smaller than the X chromosome and partially heterochromatic with a different size of C-bands. In metaphase I, they formed univalent/s in individual X0-5 with a tendency to connect with the X chromosome at early meiosis (Fig. 3a,a′), X0-7 (Fig. 3b,b′) and X0-8 (Fig. 3c,c′) or were bivalent in sample X0-12 (Fig. 3d,d′), with clearly seen differences in the intensity of hybridization signal located distally. These individuals were not examined using Ag-NO_3_ and fluorochrome staining. Additionally, a potential translocation between autosome M3 and the B chromosome^15^ was observed in representatives of the nine localities belonging to both mean races. However, no FISH cluster was revealed in this heterozygous bivalent (Table 1; Fig. 2a,a′,c,c′,d,d′,f,f′).

### Heterochromatin patterns

**Figure 4:**
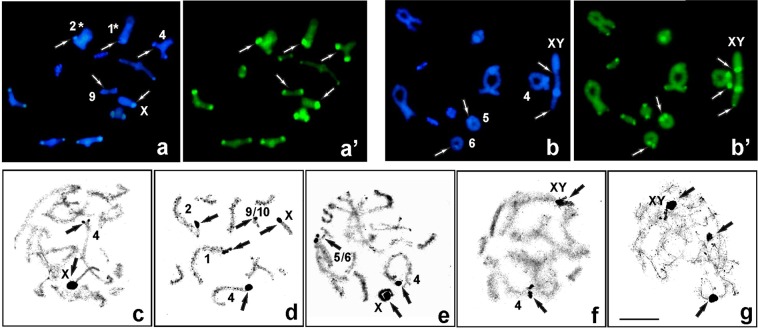
Examples of DAPI (blue) and CMA_3_ (green) stained heterochromatin in the diakinesis of individuals X0-3 (**a**,**a**′) and XY-6 (**b**,**b**′). White arrows indicate DAPI- or very weak DAPI+/CMA_3_+ bands on heteromorphic L1_,_ L2 (marked with asterisks *), homozygous M4, S9 (**a**,**a**′) and M4-M6 bivalents (**b**,**b**′); DAPI+/CMA_3_+ band in the paracentromeric region on the X chromosomes and DAPI−/CMA_3_+ on bivalent XY. Silver staining (**c**–**g**) of chromosomes in individuals X0-1 (**c**), X0-3 (**d**), Co-1 (**e**), XY-2 (**f**) and Co-1 (**g**). The arrows indicate the presence of active NORs coinciding with rDNA clusters (see Table 1). Scale bar = 10 µm.

After both C-staining and DAPI/CMA_3_ double staining, chromosome regions showed variation in constitutive heterochromatin blocks among the chromosomes in the set and the analysed individuals. All specimens had C-positive paracentromeric bands which varied in size and in rare cases distally located C-bands (Figs 2a′–j′ and 3a′–d′). Generally, thin C-bands in some autosomes were DAPI-negative (DAPI-, it was impossible to locate DAPI-positive bands) or weakly DAPI-positive (DAPI+) and were always CMA_3_ + (GC-rich). In contrast, thick C-bands (occupying the region next to the centromere) showed brightly DAPI + and CMA_3_ + signals (containing both AT- and GC-rich regions) (Fig. 4a,a′,b,b′). In such cases, DAPI+ and CMA_3_ + blocks were located close to each other, but only bright CMA_3_ + signals coincided with 18S rDNA and active NOR/s (Fig. 4c–g).

### Sex chromosome/s

**Figure 5:**
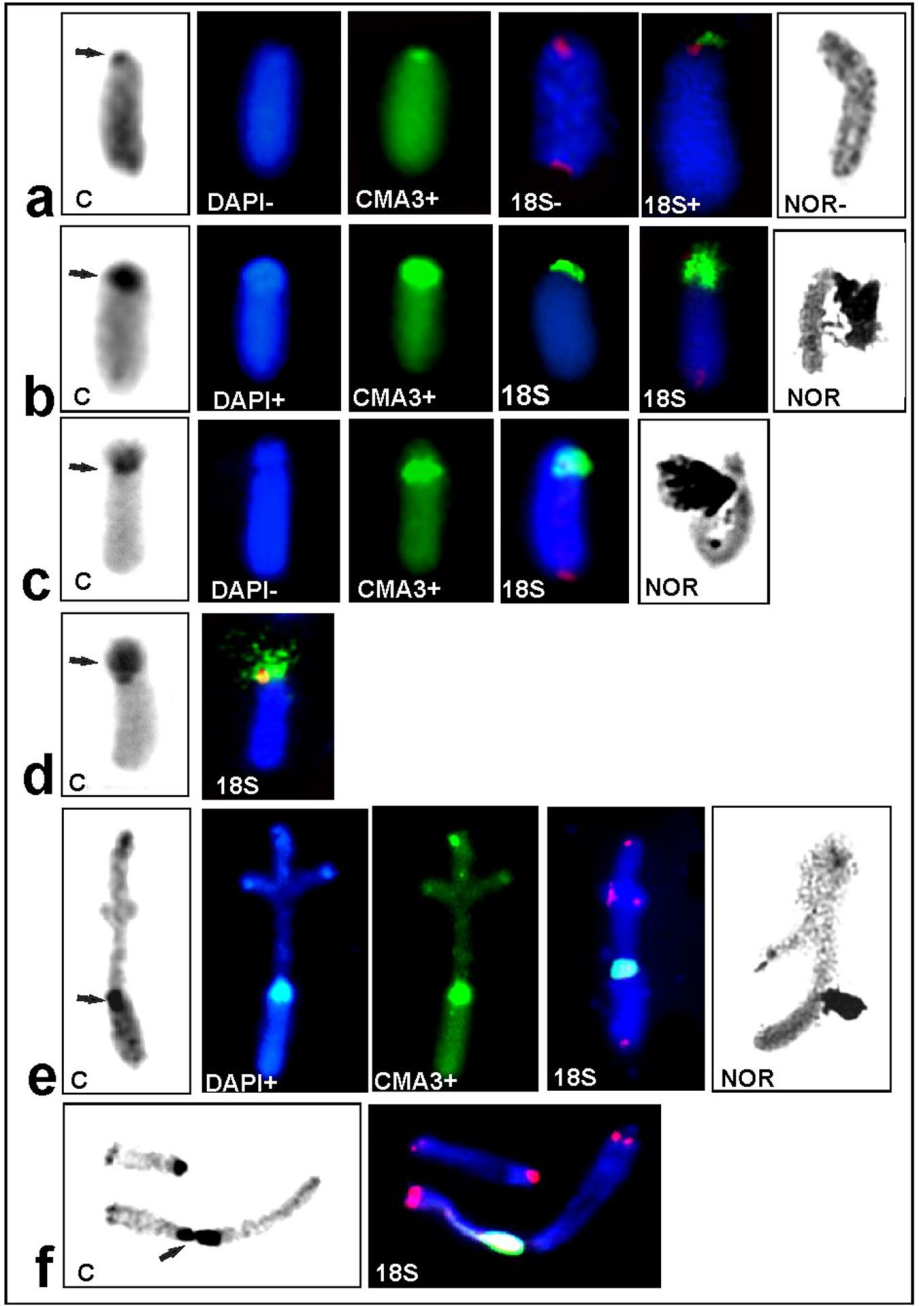
Morphotypes of the X chromosome in individuals X0-4 (**a**), X0-1 (**b**), X0-9 (**c**), X0-11 (**d**), XY-2 (**e**) and XY-8 (**f**); C-, DAPI (blue) and CMA_3_ (green) stained heterochromatin, as well as 18S rDNA (green)/telomeric DNA probes (red) and the NORs of diakinesis (**a**–**e**) and spermatogonial metaphase (**f**). In the X0/XX race, DAPI is not visible (DAPI−) as well as hybridization areas are not (18S−) or very poorly (18S+) visualized (**a**) or C/DAPI/CMA_3_ bands and FISH signals varied in size/position/intensity (**b**–**d**); this chromosome generally demonstrates a correspondence between the location of major ribosomal genes and the position of C-bands, GC-rich and NOR sites (arrows). In the neo-XY system (**e**–**f**), thick C/DAPI/CMA_3_ blocks co-localized with rDNA-FISH/NOR signals in one of the arms of neo-X (arrows).

The various banding techniques (C- and DAPI/CMA_3_ double staining, FISH with 18S rDNA, and silver staining) revealed some differences between the types of X chromosome in the X0/XX race (Fig. 5a–d). In these cases, four types were found: (1) standard acrocentric with a paracentromeric thin C-band; or (2) thick C-band; (3) subacrocentric with a euchromatic short second arm; and (4) subacrocentric with a heterochromatic short second arm (Table 1; Fig. 5a–d). In the first type, the acrocentric X chromosome has a very thin C-band, DAPI- and weakly CMA_3_+ bands; in this case, ribosomal probe FISH and NOR signals were not visible, probably due to the presence of only a few rDNA genes; i.e., less than the minimum number detectable by FISH^16^ or presented a very weak FISH signal (Fig. 5a). However, thick C-bands located on the acrocentric X chromosome contain a GC-rich band coincident with the rDNA-FISH signal and active NOR (Fig. 5b). In both types (3, 4) of subacrocentric X chromosome (with a euchromatic or heterochromatic short second arm), DAPI−/CMA_3_+ (GC-rich) signals and a large cluster of 18S rDNA located in the paracentromeric region coinciding with the active NOR was detected (Fig. 5c,d; 5d: fluorochrome-staining and NOR not shown). In the neo-XY/neo-XX race, bright DAPI +/CMA_3_+ signals (containing AT- and GC- bases) and C-bands were located in the paracentromeric region on the bi-armed neo-X (Fig. 5e,f). In diakinesis and spermatogonial metaphase, the 18S rDNA probe showed a cluster of rDNA genes located on one of the arms of neo-X which coincided with the GC-rich region and active NOR.

**Figure 6:**
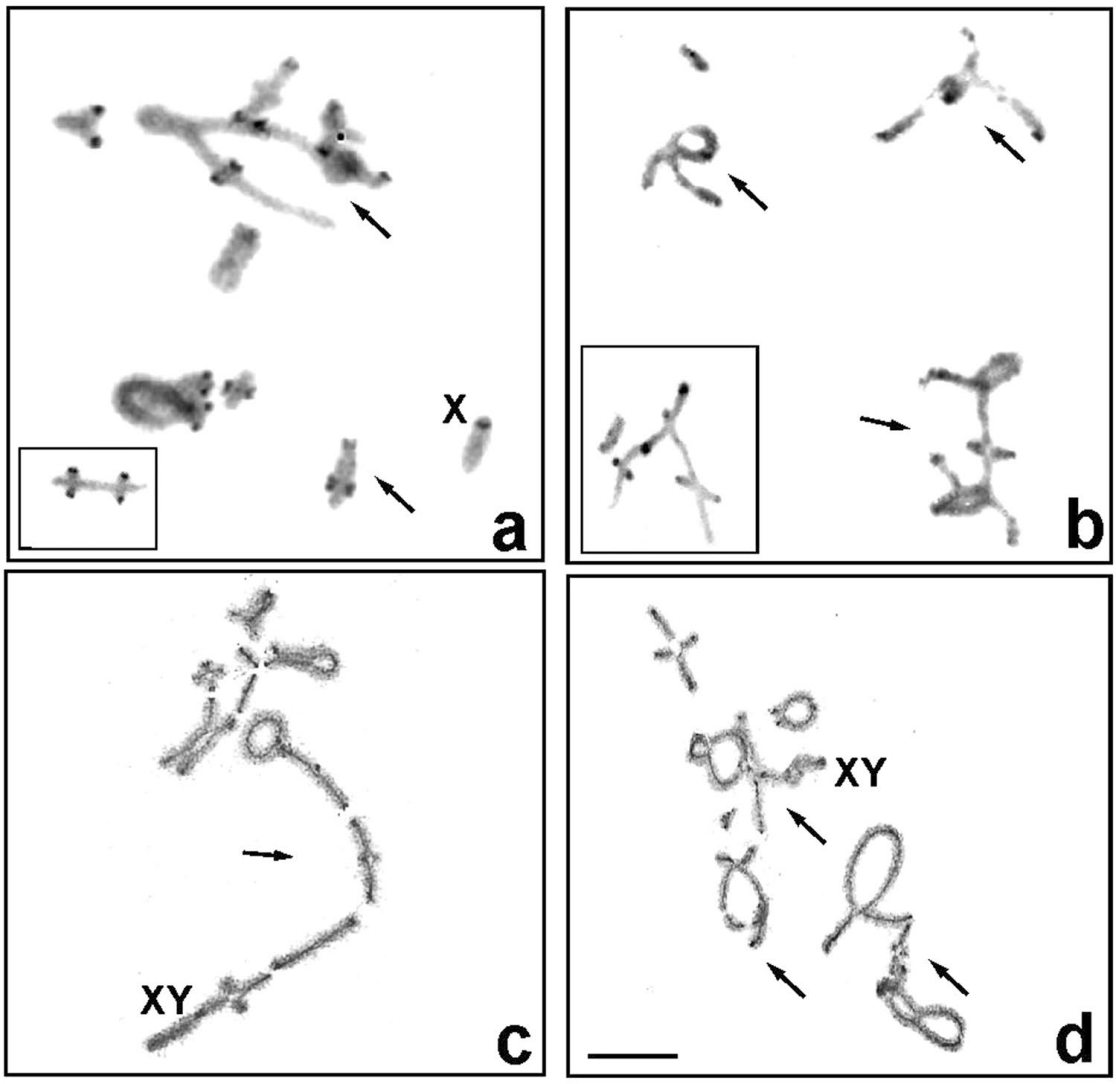
C-banding (**a**,**b**) and silver staining (**c**,**d**) at diakinesis showing chromosomal translocation caused by the rearrangement of parts between different non-homologous chromosomes in individuals X0-1 (**a**), X0-4 (**b**) and Co-1 (**c**,**d**) (arrows). Scale bar = 10 µm.

Accordingly, in most analysed individuals in both races, heteromorphism of the rDNA signals and C-banding in terms of the size and strength and the presence or absence of cluster/bands on respective homologous chromosomes in different autosomes (heterozygous in Table 1, and marked with an asterisk in relevant figures) was observed. Such polymorphism was clearly relevant to chromosomal rearrangements such as inversion and/or translocation between some of the chromosomes that were sometimes visible from pachytene to diakinesis (e.g., Figs 2a,c,e′,i,i’,j and 6a–d).

## Discussion

In the present study, we analysed the extent and distribution of cytogenetic variability in the populations of *P. sapporensis* including two chromosomal races. This species provides a non-trivial opportunity to study the mechanisms underlying speciation. It should be noted that the chromosomal rearrangements may reinforce a barrier to gene flow between the races as it was demonstrated by Searle *et al*.^17^. The resolution of physical mapping depends on the degree of chromosomes condensation. The meiotic divisions are better than the mitotic owing to a lesser degree of chromosome condensation and a clear distribution pattern of heterochromatic regions. Generally, our findings revealed extensive variation in the number and locations of the 18S rDNA gene between and within races/subraces/localities around the contact zone of the two chromosomal races. FISH with an rDNA probe showed: (1) from two to five sites on the medium bivalent and the X chromosome in the X0/XX race^2^; (2) two clusters on the medium bivalent and neo-X in the neo-XY/neo-XX race; and (3) from one to five rDNA signals in medium and rarely in long-sized autosomes and sex chromosomes (X and neo-X) in the X0/XX-Naganuma/Yotei and XY/XX-Tanno/Oketo subraces. Additionally, a high level of heterozygosity (i.e., the occurrence of rDNA loci only on one of two homologous chromosomes) in different localities was observed (Table 1). Consequently, no obvious association between the number and heteromorphism of rDNA loci and the chromosomal races was observed. Chromosomal localization of ribosomal genes in this species has been associated with C-positive regions, GC-rich heterochromatin and active NORs, similar to most of the cases in katydid species (e.g.,)^18^,^19^. Extensive polymorphism in the number and localities of 18S rDNA loci on autosomes and sex chromosome/s among grasshopper species can be also seen in the closely related species *Podisma pedestris* (Linnaeus, 1758)^20^ and to a lesser extent in other Podismini^21^. Differences in the number of rDNA/NORs loci with variable positions are also a peculiar characteristic in some katydids (Phaneropterinae: Barbitistini), involving populations or groups of species that have diverged recently and probable hybrid populations^18^,^19^,^22^.

The individuals examined in this study also showed differences in the intensity and position of the hybridization signals of the (TTAGG)_*n*_ probes in both autosomes and sex chromosomes. Variation in the intensity of signals in chromosomes, including sex chromosomes, is presumably due to the presence of different numbers of telomeric repeats. The lack of tDNA-FISH signals in some chromosomes may suggest a low number of copies of telomeric repeats^23^. Some chromosomes possessed additional hybridization signals in interstitial positions, indicating the presence of ITSs which are related to the telomere-telomere fusions of the chromosomes, inversions, unequal crossing over, or the insertion of telomeric DNA into unstable sites during the repair of double-strand breaks^24^. ITSs are associated with constitutive heterochromatin and could represent good markers of chromosomal rearrangements such as inversion or translocation (e.g.)^25^.

From a karyological point of view, the variation in the number and positions of 18S sites, heteromorphism in the size of the cluster in homologous chromosomes and the presence of additional interstitial sites of the (TTAGG)_*n*_ sequences in individuals/populations may indicate that they are still in a unstable period. Heteromorphism of rDNA clusters and C-banding were observed in most of analysed individuals.

The occurrence of the polymorphism of B chromosomes (variation in size, morphology and C-banding content) has previously been noted in *P. sapporensis* populations^15^. In the present data, only one morphotype (metacentric B_5/6iso_ variant) of the supernumerary chromosomes (Bs) has been found in both races, suggesting that they had originally occurred in the ancient *P. sapporensis* population prior to the establishment of different chromosomal races^10^. Additionally, we found another type of B chromosome — acrocentric B_n_ types (likely new or heteromorphic B_1_ and B_2_ types), in four populations of the X0/XX race (X0-5, X0-7, X0-8 and X0-12). The distribution of both B types and translocation between B and autosomes (M3 pair) are probably not related to the chromosomal rearrangements.

It should be noted that rDNA-FISH analysis revealed signals on the sex chromosome in both the X (X0/XX) and neo-X (neo-XY/neo-XX). Polymorphism of C-heterochromatin contains a single (except the X with very thin C-bands) probably paracentromeric rDNA loci (different in size) involving the X chromosome in the X0/XX race (Fig. 5), confirming a dynamic karyotypic evolution. The X chromosome with the rDNA cluster originated independently of the chromosomal rearrangements. In sex chromosomes in the neo-XY/neo-XX race, it is likely that the neo-Y lacks rDNA because a single rDNA cluster was observed only in the paracentromeric region/proximally located just within the metacentric neo-X.

The *Podisma* grasshopper is a geographically diverse species, comprising two major chromosomal races: western (X0/XX) and eastern (neo-XY/neo-XX). The western populations were found to have a higher genetic variability compared to the eastern region^10^. Our study clearly demonstrates the existence of differentiation in the number and location of 18S rDNA genes, as well as the pattern of constitutive heterochromatin among and within both chromosome races, a result consistent with previous studies^7,8,10,15^. However, the hybrid individuals may have intermediary patterns resulting in heterozygous markings (e.g. X0-4 and XY-4).

In summary, the present study has focused on the cytogenetic mapping of rDNA coding genes and telomeric sequences in *P. sapporensis*. Variability in the karyotype organization was observed for heterochromatin and 18S rDNA clusters. Karyotypic differences between races show that chromosomal divergence occurred during their speciation. The use of molecular tools with this species as a model will enable a better explanation of the processes involved in the origin and differentiation of the different races and probable hybridization in *Podisma* species. Further karyotype analyses by FISH rDNA genes, telomeric sequences and other genetic chromosomal markers should be performed in other individuals and populations. Also, comparative studies from the free borders of this species distant from the interracial contact zone are important.

## Material and Methods

### Taxon sampling

In this study, new samples were analysed from the neighbourhood of hybrid population (Mikuni Pass population). A total of 165 males of *P. sapporensis* from 22 localities in Hokkaido collected in the year 2015 were studied cytogenetically as described in the following sections. These samples covered both X0/XX and neo-XY/neo-XX chromosomal races, including hybrid populations of these races (Table 1, Fig. 1).

### Chromosome preparation

Chromosome preparations were produced from male gonads. The grasshoppers were dissected and the testes were fixed in ethanol:acetic acid (3:1). Slides were prepared by squashing testis follicles in one drop of 45% acetic acid and covering them with coverslips that were removed after freezing on a block of dry ice (frozen carbon dioxide) for a few minutes. Slides were first examined under a phase-contrast microscope to check for the availability of meiotic divisions and the quality of chromosome spreads.

### Chromosome staining

For karyotyping and the identification of chromosome rearrangements, the preparations from all specimens were used for C-banding according to Sumner^26^. Additionally, some slides were analysed qualitatively by CMA_3_ (chromomycin A_3_) and DAPI (4,6-diamidino-2-phenylindole) staining^27^ as well as active nucleolus organizing regions (NORs)^28^.

### Fluorescence *in situ* hybridization (FISH)

For the 42 males selected from 22 localities (including five specimens collected in 2005 and 2007 from two localities), the best preparations were used for fluorescence *in situ* hybridization. FISH was carried out as described previously^21^ using an 18S rDNA probe from orthopteran labelled through PCR with biotin-16-dUTP (Roche Diagnostics GmbH, Germany). A probe from the telomeric DNA sequence (TTAGG)_*n*_ was generated by PCR in the absence of a template, using TTAGG_F (5′- TAA CCT AAC CTA ACC TAA CCT AA-3′) and TTAGG_R (5′-GGT TAGGTT AGG TTA GGT TAG G-3′)^29^ as primers. The visualization of hybridized DNA labelled with biotin-16-dUTP or digoxigenin-11-dUTP (Roche, Diagnostics GmbH, Germany) was performed with avidin-FITC (Invitrogen, USA) or anti-digoxigenin rhodamine (Roche Diagnostics GmbH, Germany), respectively. Digital images were obtained using a CCD DS-U1 camera coupled to fluorescence microscope. The software NIS-Elements BR2 was used for camera control and the merging of DAPI and fluorochrome images of the paints.
